# Upregulation of Lysyl Oxidase Expression in Vitreous of Diabetic Subjects: Implications for Diabetic Retinopathy

**DOI:** 10.3390/cells8101122

**Published:** 2019-09-21

**Authors:** Manju L. Subramanian, Thor D. Stein, Nicole Siegel, Steven Ness, Marissa G. Fiorello, Dongjoon Kim, Sayon Roy

**Affiliations:** 1 Department of Ophthalmology, Boston University School of Medicine, Boston, MA 02118, USA; Manju.Subramanian@bmc.org (M.L.S.); Nicole.Siegel@bmc.org (N.S.); steven.ness@bmc.org (S.N.); Marissa.Fiorello@bmc.org (M.G.F.); 2 Department of Pathology and Laboratory Medicine, Boston University School of Medicine, Boston Medical Center, Boston, MA 02118, USA; tdstein@bu.edu; 3 Department of Veterans Affairs (VA) Medical Center, Bedford, MA 01730, USA; 4 VA Boston Healthcare System, Boston, MA 02130, USA; 5 Department of Medicine, Boston University School of Medicine, Boston, MA 02118, USA; djkim@bu.edu

**Keywords:** lysyl oxidase, hyperglycemia, diabetic retinopathy, retinal lesions

## Abstract

Animal studies have shown diabetes-induced lysyl oxidase (LOX) upregulation promotes blood-retinal-barrier breakdown and retinal vascular cell loss associated with diabetic retinopathy (DR). However, it is unclear whether changes in LOX expression contribute to the development and progression of DR. To determine if vitreous LOX levels are altered in patients with DR, 31 vitreous specimens from subjects with advanced proliferative DR (PDR), and 27 from non-diabetics were examined. The two groups were age- and gender-matched (57 ± 12 yrs vs. 53 ± 18 yrs; 19 males and 12 females vs. 17 males and 10 females). Vitreous samples obtained during vitrectomy were assessed for LOX levels using ELISA. LOX was detected in a larger number of PDR subjects (58%) than in non-diabetic subjects (15%). Additionally, ELISA measurements showed a significant increase in LOX levels in the diabetic subjects with PDR, compared to those of non-diabetic subjects (68.3 ± 112 ng/mL vs. 2.1 ± 8.2 ng/mL; *p* < 0.01). No gender difference in vitreous LOX levels was observed in either the diabetic or non-diabetic groups. Findings support previous reports of increased LOX levels in retinas of diabetic animals and in retinal vascular cells in high glucose condition, raising the prospect of targeting LOX overexpression as a potential target for PDR treatment.

## 1. Introduction

Diabetic retinopathy (DR) is the leading cause of vision impairment and blindness among working-age adults [1]. The early stages of diabetic retinopathy are marked by the development of thickened capillary basement membrane (BM), formation of acellular capillaries and pericyte ghosts, and the clinical manifestation of vascular hyperpermeability in the retina [2,3,4]. A critical function of the BM is its ability to act as a selective permeable barrier, and diabetes-induced alteration of its ultrastructure is known to promote vascular permeability [5]. Several studies have indicated that changes in the structural integrity of the BM can have profound detrimental effects with respect to its functionality [6,7]. While excess synthesis of BM components contributes to BM thickening, lysyl oxidase (LOX), a key enzyme responsible for the maturation and development of BM, has been implicated in regulating the ultrastructural integrity of the BM [8]. The functionality of the thickened BM is compromised by high glucose (HG)-induced LOX upregulation and increased activity, which leads to breakdown of blood-retinal-barrier (BRB) characteristics associated with diabetic retinopathy [9].

In addition to its role in the maintenance and stabilization of the BM, LOX has been shown to possess pro-apoptotic effects [10,11,12,13,14,15]. Retinal vascular cells are known to die by apoptosis in diabetic retinopathy and undergo accelerated cell loss, leading to formation of pericyte ghosts and acellular capillaries, which can not only contribute to vascular changes in early stage of diabetic retinopathy, but also to neovascularization in PDR [16]. A recent study from our lab has shown that HG- or diabetes-induced LOX upregulation promotes apoptosis in retinal vascular cells, and that normalization of LOX overexpression using a siRNA strategy in vitro or through a LOX heterozygous knockout animal model in vivo prevented apoptosis [17]. These data suggest that LOX overexpression in the retina may contribute to retinal vascular lesions seen in diabetic retinopathy.

Interestingly, LOX is known to play an integral role in regulating fibrosis [18,19,20], which is associated with the progression of PDR [21,22], and characterized by excess accumulation of extracellular matrix. HG or diabetes is known to induce excess extracellular matrix synthesis [23,24,25,26,27] and upregulate LOX [9,17], which could lead to accumulation and stiffening of the extracellular matrix [7].

Several studies using cell culture and animal models of diabetes indicate that HG or diabetes significantly increases the expression and activity of LOX in renal, pulmonary, and retinal tissues [9,28,29,30]. In patients with diabetes, increased LOX activity has been reported in skin biopsies and vitreous samples from the eye [31,32,33] as evidenced from increased LOX-mediated collagen crosslinks and early glycation products, glucitolyllysine and glucitolylhydroxylysine, which are indirect measures of LOX activity [32,33]. Our current findings relate to assessment of LOX levels directly in the vitreous of eyes from diabetic and non-diabetic individuals. As mounting evidence suggests increased LOX activity in diabetes, little is known about LOX levels in the diabetic vitreous. While studies have shown that cytokines and growth factors, such as VEGF, are increased in the vitreous of PDR patients and play a critical role in the pathogenesis of PDR, the involvement of LOX upregulation in this process is not well understood. In this study, we examined whether LOX levels were altered in vitreous samples freshly isolated from diabetic subjects with advanced PDR and non-diabetic subjects.

Overall, studies conducted with in vitro cell culture models and animal models of diabetes suggest that excess LOX is likely involved in the pathogenesis of diabetic retinopathy. Hence, in this study, we investigated whether patients with advanced diabetic retinopathy expressed altered levels of LOX in the vitreous humor, compared to those of non-diabetic subjects.

## 2. Materials and Methods

This prospective observational study involving human subjects was conducted at Boston Medical Center, and adheres to the Declaration of Helsinki. Institutional review was approved, and signed informed consent was obtained for all subjects enrolled in the study. Patients were eligible for study participation if they were greater than 18 years of age, and were scheduled for vitrectomy surgery in at least one eye as part of standard care. Once enrolled, clinical and demographic data were obtained through patient questionnaire and electronic medical record review. A total of 58 vitreous specimens were obtained during vitrectomy surgery for various vitreoretinal conditions. In patients with diabetic retinopathy, the indications for surgery included vitreous hemorrhage and tractional retinal detachments. In non-diabetic subjects, indications for vitrectomy included rhegmatogenous retinal detachments, epiretinal membranes, and macular holes. With regard to additional therapies, 12 PDR patients received antiangiogenesis therapy, 21 PDR patients received panretinal photocoagulation (PRP), and 11 PDR patients had fibrotic membranes. These are not surprising, since PRP and anti-VEGF therapy are the mainstay of treatment for patients with PDR. PDR is defined by the presence of active neovascularization, and there were signs of this in all of our PDR patients.

At the start of the vitrectomy procedure, the surgical team placed an infusion line into the vitreous. Then 0.5–1.0 mL of undiluted vitreous fluid was aspirated through the vitrectomy probe into an attached, sterile 3-milliliter syringe. Once the undiluted vitreous specimen was collected, saline infusion was immediately turned on to allow fluid to flow into the vitreous cavity to re-pressurize the eye. After the collection of the vitreous sample, the entire operation for each study participant proceeded as it would under the standard of care. Biologic samples removed from the eye were placed in tubes labeled with a coded number non-identifiable to the source, and immediately centrifuged for 15 min at 12,000 rpm to separate the cellular contents, and the supernatant was aliquoted at 100 μL, frozen and stored at −80 °C. At the time of assay, frozen vitreous fluid was thawed, diluted 1:30 with phosphate buffered saline, and then centrifuged at 17,000 g at 4 °C for 10 min. The supernatant was measured for total LOX protein levels using a colorimetric LOX enzyme-linked immunosorbent assay (LifeSpan Biosciences, Seattle, WA, USA).

## 3. Results

**Figure 1:**
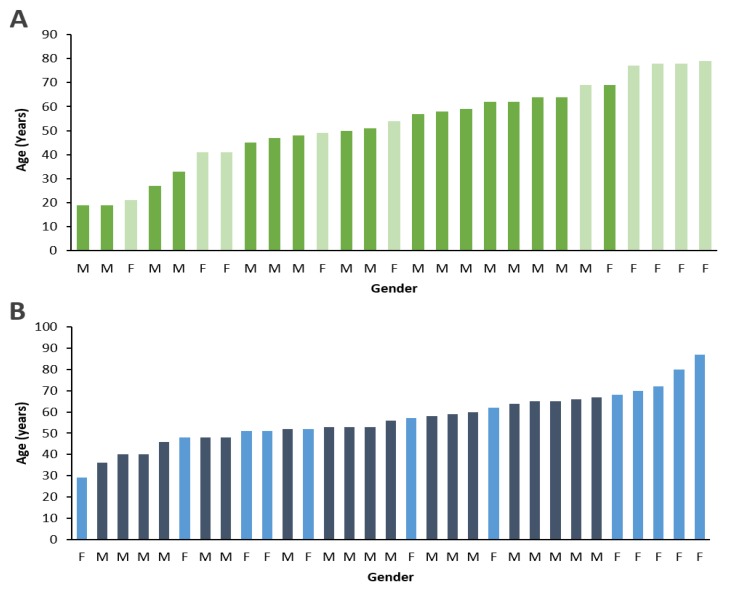
Age and gender distribution of study subjects. Demographic data shown in graphs indicate that (**A**) non-diabetic subjects and (**B**) diabetic patients with advanced PDR were age-matched and gender-matched. Dark shade = Male; Light shade = Female.

**Figure 2:**
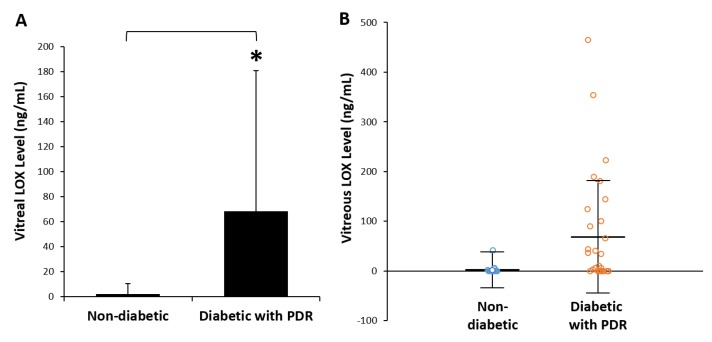
LOX levels in the vitreous humor of non-diabetic patients and PDR patients. (**A**) Graphical data illustrate that LOX level is significantly higher in the vitreous samples of PDR patients compared to those of non-diabetic patients. (**B**) To allow visualization of vitreous LOX levels, individual data points are depicted. *p < 0.01, n = 27 non-diabetic; n = 31 diabetic with PDR. Data are reported as mean ± SD; a Mann-Whitney test was used to analyze data. Data with values of *p* < 0.05 were considered significant.

Thirty-one patients with advanced PDR, and twenty-seven individuals without diabetes were recruited in this study. Individuals from both diabetic and non-diabetic groups were age-matched (57 ± 12 yrs vs. 53 ± 18 yrs, respectively), and gender-matched (19 males and 12 females vs. 17 males and 10 females, respectively; Figure 1). Assessment of total protein in the vitreous samples showed a significant increase in LOX levels in diabetic subjects with advanced PDR compared to those of non-diabetic subjects (68.3 ± 112 ng/mL vs. 2.1 ± 8.2 ng/mL; *p* < 0.01; Figure 2). Additionally, the frequency of diabetic patients with advanced PDR that had detectable levels of LOX in the vitreous was markedly increased compared to nondiabetic subjects (58% vs. 15%, respectively). There was no difference in the LOX levels of vitreous samples derived from male and female subjects in both the diabetic group and the non-diabetic group. Of note, our recent study indicated that mannitol used as osmotic control showed no effects on LOX levels in retinal endothelial cells in vitro [34].

## 4. Discussion

The results from this study provide evidence that LOX level is significantly increased in the vitreous of diabetic patients with advanced PDR compared to those of non-diabetic subjects. No significant difference in vitreous LOX levels was observed between male and female subjects for both diabetic and non-diabetic groups. The data supports our previous findings, showing high glucose or diabetes upregulates LOX expression in retinal endothelial cells in vitro, and in vascular cells of retinal capillaries from diabetic rats and mice [9,17], and that upregulation of LOX promotes cell monolayer permeability [9]. Overall, findings from the current study suggest that retinal LOX overexpression may be associated with the development of PDR.

To the best of our knowledge, this is the first study that suggests increased LOX levels in the vitreous may be associated with the development of diabetic retinopathy. Additionally, findings suggest that a consequence of LOX upregulation is compromised BRB and subsequent retinal vascular permeability [35]. Such vascular leakage of proteins and lipids in the retina can osmotically draw fluid from the intravascular space in the retinal blood vessels into the extracellular space of retinal cell layers, and contribute to clinically detectable changes, such as diabetic macular edema. Prior studies in human subjects have shown an increase in the activity of LOX in diabetic conditions [31,32,33]. However, a study comparing vitreous specimens from patients with PDR to vitreous specimens taken post-mortem from non-diabetic subjects showed decreased LOX activity in the PDR group [36]. This may be in part due to the difference in the vitreous specimen source, as reported in studies investigating post-mortem effects on biological samples, in which differential gene expression immediately following death was observed [37,38]. Our current study, alternatively, was conducted using freshly obtained vitreous samples for both diabetic and non-diabetic groups. Coral et al. also included patients with rhegmatogenous retinal detachments in their study group, a disorder that is non-vascular and mechanical in etiology [39], and this may have contributed to their finding of decreased LOX activity.

While our current study identified increased LOX level in the vitreous of diabetic patients with advanced PDR, not enough sample amount was available to assess the activity of LOX, a limitation of the study that requires further investigation. Furthermore, at this point, it is unclear why a small number of diabetic patients with advanced PDR showed low or undetectable levels of LOX in their vitreous, and conversely, why some of the non-diabetic subjects without PDR showed LOX in their vitreous. Although additional factors independent of LOX may be involved in promoting the pathogenesis of PDR, our current findings are indicative of the importance of LOX as a mediator in the development and progression of DR.

The vitreous humor serves as a stagnant reservoir for inflammatory and neoplastic mediators that occur in the retina and other parts of the eye [40]. Further studies are needed to determine if vitreous sampling for LOX could be useful as a biomarker and aide in identifying progression of diabetic retinopathy status. Findings from the current study highlight the relevance of LOX upregulation in human diabetic retinopathy. Moreover, it provides a basis for further investigation to determine if inhibiting LOX overexpression may be a useful strategy for preventing retinal vascular lesions associated with the pathogenesis of diabetic retinopathy.
